# Breath biopsy biomarkers: cell-free nucleic acids in exhaled breath condensate

**DOI:** 10.3389/fmed.2025.1693114

**Published:** 2025-10-24

**Authors:** Ondrej Pös, Jakub Styk, Monika Kubanova, Silvia Bokorova, Gergely Buglyo, Beata Soltesz, Peter Lukasz, Vladimir Benes, Vanda Repiska, Balint Nagy, Tomas Szemes

**Affiliations:** ^1^Comenius University Science Park, Bratislava, Slovakia; ^2^Geneton Ltd., Bratislava, Slovakia; ^3^Faculty of Medicine, Institute of Medical Biology, Genetics and Clinical Genetics, Comenius University, Bratislava, Slovakia; ^4^Department of Medical Genetics, Faculty of Medicine, University of Debrecen, Debrecen, Hungary; ^5^Department of Surgery, Transplantation and Gastroenterology, Semmelweis University, Budapest, Hungary; ^6^European Molecular Biology Laboratory, Heidelberg, Germany; ^7^Department of Molecular Biology, Faculty of Natural Sciences, Comenius University, Bratislava, Slovakia

**Keywords:** breath biopsy, exhaled breath condensate (EBC), cell-free nucleic acids, lung cancer biomarkers, liquid biopsy, obstructive lung diseases

## Abstract

Exhaled breath condensate (EBC) has emerged as a promising, organ-specific biofluid for non-invasive molecular diagnostics. While breath analysis has traditionally focused on volatile organic compounds (VOCs), recent advances have shifted attention toward non-volatile constituents, particularly cell-free nucleic acids (cfNAs) such as genomic DNA, mitochondrial DNA, mRNA, miRNA, long non-coding RNA, and microbial genetic material. These molecules reflect the respiratory tract biology and can serve as biomarkers for a range of clinical conditions, including lung cancer, obstructive lung diseases, infections, and potentially even systemic disorders. This review summarizes the current knowledge on cfNAs in EBC, highlighting technical challenges in sample collection and nucleic acid extraction. We provide a comparison of EBC collection devices, discuss optimization strategies for nucleic acid recovery, and examine emerging applications such as early cancer detection, treatment monitoring, infection diagnostics, and endotyping of chronic airway diseases. The feasibility of at-home EBC sampling with portable collection devices offers additional advantages, potentially overcoming logistical and psychological barriers that often delay clinical care. Although limitations remain, including low cfNA yield and lack of standardization, ongoing innovation in sampling and molecular techniques is rapidly expanding the translational potential of breath biopsy. With further development, EBC-based cfNA profiling may complement blood-based liquid biopsies and, in specific contexts such as lung cancer, provide additional organ-specific information.

## 1 Introduction

Exhaled breath sampling to analyze disease-associated changes is a long-established concept. It has been used to recognize certain diseases for centuries. For example, a sweet breath odor was associated with diabetes mellitus, a fishy odor with liver disease, and a urine-like odor with kidney disease (1). The basics of modern breath analysis came from the early 1970s when Linus Pauling detected 250 volatile organic compounds (VOCs) in human breath samples (2). So far, thousands of VOCs have been identified to be exhaled with a gas-phase fraction of breath (3), constituting a unique ‘fingerprint' that reflects the physiological status of an individual. Nowadays, several breath-based tests utilizing endogenous or exogenous volatile compounds have been established (4), and novel technologies with increased emphasis on metabolomics have given rise to “breathomics”, a field that provides valuable insight into the status of various disease-related metabolic pathways by quantifying the VOCs (5).

**Figure 1:**
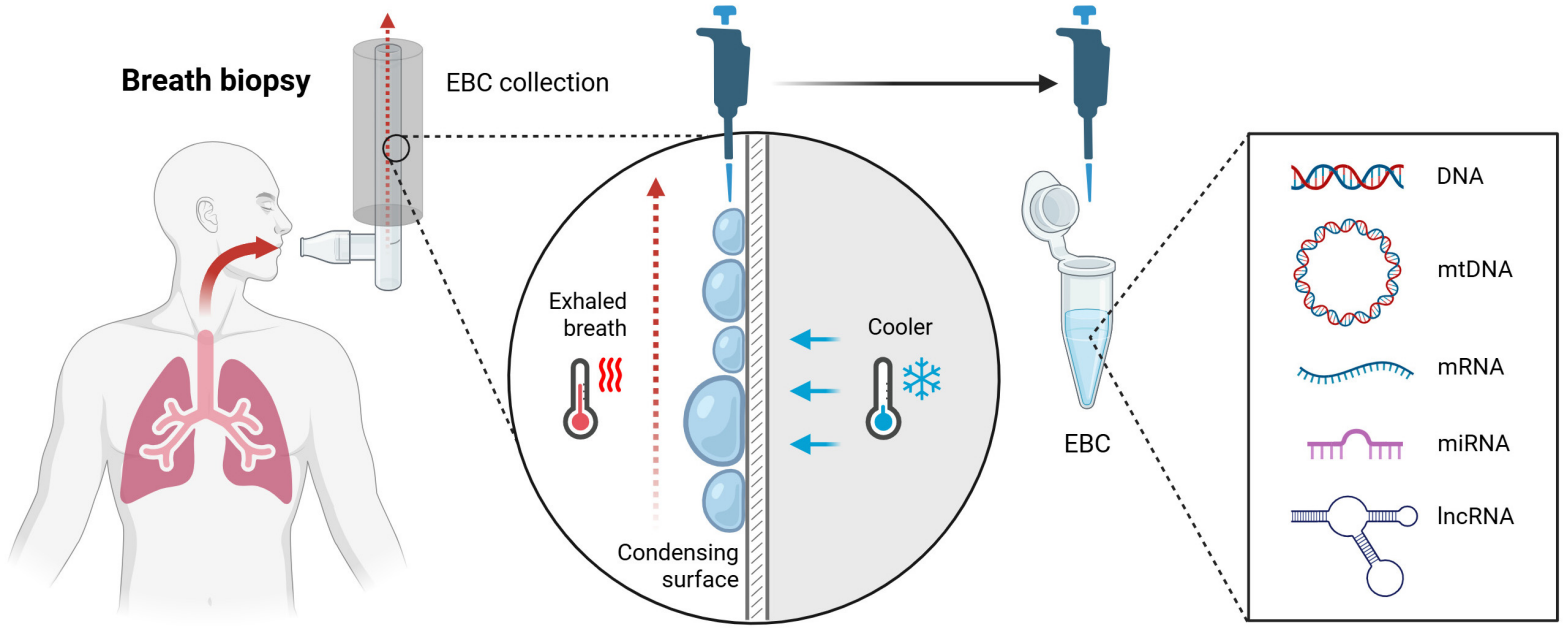
The scheme of EBC collection using the RTube device as an example. Warm exhaled breath passes through a mouthpiece into a pre-cooled condenser. Upon contact with the chilled surface (maintained by a cooling sleeve or integrated cooler), water vapor and respiratory aerosols form microdroplets that merge and drain into a collection tube. The resulting exhaled breath condensate (EBC) contains numerous constituents, including cell-free nucleic acids (cfDNA, cfRNA) of host and/or microbial origin, suitable for downstream molecular analyses with biomedical potential. Figure was created with BioRrender.com.

In addition to volatile compounds, exhaled breath contains respiratory droplets that may carry non-volatile matter such as microbiota (e.g., bacteria, fungi, and viruses) (6), residues of cells, electrolytes, sugars, enzymes, and nucleic acids (7). These aerosol particles of various sizes (1–1,000 μm) originate from the respiratory tract, thus providing non-invasive access to the genetic material of the lung epithelial lining (Figure 1) (8). However, the low yield of DNA in breath samples has challenged the concept of identifying genomic alterations for screening or diagnostic applications. Only the development of systematic approaches to exhaled breath collection and nucleic acid extraction (9), with the introduction of powerful genomic techniques, could promote this non-invasive biopsy to the next level. Despite the demonstration of exhaled breath-derived DNA potential to reveal early neoplastic changes or indicate apoptosis eliminating damaged cells with altered genetic material, most research interest still focuses on VOCs analysis, thus leaving the non-volatile compounds an understudied biomarker with barely tapped potential in biomedical applications.

We conducted a literature search in PubMed to identify original and review articles published between January 2004 and January 2025. The search strategy used the terms (“exhaled breath condensate”) AND (“DNA” OR “RNA” OR “nucleic acids”). Titles and abstracts were manually screened for relevance. Eligible records included: (i) Original research articles reporting cell-free nucleic acids (cfNAs) in human EBC samples, including genomic DNA, mitochondrial DNA, mRNA, microRNA (miRNA), long non-coding RNA (lncRNA), and microbial nucleic acids; (ii) Review articles that provided critical synthesis or contained particularly valuable conclusions relevant to clinical or methodological aspects of EBC-based analysis. We excluded purely VOC-focused studies, animal studies, and papers lacking cfNA data. From the selected articles, we abstracted information on study design, sample size, collection device, extraction method, analytic platform, and principal outcomes, which informed the synthesis tables and discussion presented in this review.

## 2 Exhaled breath condensate

The principle of breath biopsy sampling is the guidance of exhaled breath through a cooling system where the gas phase condenses into a liquid, known as exhaled breath condensate (EBC) (27). Various EBC collection devices have been used in studies, whether custom-made or commercially available (Table 1), such as ECoScreen devices, including ECoScreen 1 (Jeager, Germany), ECoScreen 2 (FILT Lungen-& Thorax Diagnostik GmbH, Germany), ECoScreen Turbo (VIASYS Healthcare, Germany), RTube (Respiratory Research Inc., USA), Turbo-DECCS System (Medivac Srl., Italy), and Anacon (Biostec, Spain), which are comprehensively described in reviews (13, 16, 28). This also includes wearable devices for real-time monitoring of EBC biomarkers, such as the EBCcare smart mask (25). Mask-based platforms are at the forefront of innovation as they seamlessly integrate without additional burden on users and are equipped with miniature condensers for *in situ* collection and analysis. This technology offers the potential for continuous and non-invasive health monitoring directly from the breath, paving the way for personalized medicine by moving diagnostics from centralized laboratories to daily life (29).

**Table 1:** Overview of EBC collection devices and their properties across different clinical and research scenarios.

**Device**	**Method**	**Pros**	**Cons**	**Ref**.
RTube collector^1^	Passive condensation	Portable, rapid cooling, disposable, no cleaning required, allows self-collection	Pre-cooling required, low efficiency (long collection time), unintended thawing of the EBC, waste generation	(10–12)
ECoScreen (1/2 Turbo)^1^	Active condensation	No pre-cooling required, variable volume of collected EBC	Lack of control over the condensing temperature (ECoScreen 1), power required, time-consuming clean up	(13, 14)
Turbo-DECCS System^1^	Active condensation	Separation of dead space and alveolar air^3^, digital thermostat, compatible with mechanical ventilation	Power required, non-disposable (time-consuming clean up), limited validation	(11, 15)
Anacon^1^	Active condensation	Direct integration with ventilator circuit, glass condensing surface increases efficiency	Limited validation, for use on ventilated patients only, warm-up during collection, non-disposable (time-consuming clean up)	(16, 17)
Khorshid's device^2^	Passive condensation	Low consumable costs (1–2 EUR per sample), collection of frozen EBC, inert material (stainless steel)^4^	Long pre-cooling required, limited collection duration^5^, increased exhalation resistance, freezing EBC inside the tubes, not yet validated, time-consuming clean up	(11)
Glass EBC condenser^2^	Passive condensation	Suitable for mitochondrial (mtDNA) analysis	Pre-cooling required, low DNA yields, time-consuming clean up	(18–20)
Jacketed cooling borosilicate pipe EBC condenser^2^	Active condensation	Suitable for long non-coding RNA (lncRNA) analysis	Advanced cooling required (cryostat), complex collection setup, lack of standardization, not yet validated, time-consuming clean up	(8, 21, 22)
Jouyban's Breath Sampling Setup^2^	Passive condensation	NA	NA	(9)
PTFE and parafilm device^2^	Passive condensation	Low consumable costs (0.5 USD per sample), disposable hydrophobic film and sterile straw (no cleaning required)	Ultra-low temperature pre-cooling required (−70 °C), no control of saliva contamination, not yet validated	(6)
Mask-based EBC collector^2^	Passive condensation	Wearable, self-collection, reduced contamination risk	Pre-cooling required, limited validation, long collection time, person-dependent efficiency, waste generation	(23, 24)
EBCcare smart mask^2^	Passive condensation	Real-time and remote monitoring, integrated self-cooling system, power-free, estimated low consumable costs (1 USD per sample)	Proof-of-concept, not yet validated	(25, 26)

For detailed study performance data, see Supplementary Table 1.

^1^Commercially available; ^2^Custom-made devices; ^3^Includes an AAV function to help discriminate the alveolar portion of exhaled air; ^4^Prevents denaturation of protein biomarkers; ^5^Max. 6 min recommended to avoid thawing.

The design of collection equipment differs in several methodological options to prevent salivary contamination, inhalation through the condenser, and contamination by ambient air. Other important aspects are the material and area of the condensing surface, cooling method, and condensation temperature that may affect the final volume and composition of the EBC sample (30). Moreover, portable EBC collection devices enable at-home sampling, potentially overcoming logistical and psychological barriers that delay clinical visits and hinder early cancer diagnosis.

### 2.1 Prior to EBC collection

Even though there is inter-individual variability in EBC volume, the significant effect of age, gender, airway disease status (31), or even moderate-intensity exercise before sampling (32) on EBC yield has not been confirmed. On the other hand, physiological aspects and activities of the subject prior to collection may affect the composition of the sample. The effect of physical activity, cigarette smoking, and drinking caffeinated or carbonated beverages has been demonstrated (13), so it is recommended for subjects to refrain from exercise, smoking, eating, and drinking 1–3 h before EBC collection. However, the vast majority of studies assessed volatile compounds, and thus, the effect on cfNAs content remains unexplored.

### 2.2 EBC collection

Since the main target of EBC analysis is compounds originating from the lower respiratory tract, sample contamination by saliva should be avoided (13). Various recommendations have been described, including mouth washing with sodium bicarbonate 4.5% prior to sampling, periodic swallowing of the accumulated saliva during collection, and prevention of forced expiration (33), but the standard solution is a saliva trap or saliva filter applied to the collection device. Contamination by the ambient air is usually avoided by a one-way valve or a two-way non-rebreathing valve (34) that also prevents subjects from inhaling cold air that has passed through the condenser (30).

Various approaches can be used for breath cooling, including dry ice, wet ice, cooling sleeves, liquid nitrogen, and electrical refrigerating systems (35), so the condensation temperature ranges from 4 °C to −80 °C between studies. It has been found that lower temperature increases EBC collection rate (28), but practical aspects should also be considered, such as the common technical infrastructure of the sample collection facility, or even equipment allowing at-home collection that may not allow to reach ultra-low temperatures. Moreover, temperature may also affect the concentration of different compounds (36), but the optimal temperature to enrich EBC for nucleic acids remains unclear.

The surface of condensing apparatus is another important factor affecting the yield of EBC and biomarkers. A larger surface area is able to harvest more EBC for a given time, which is why some custom-made devices (32, 37) have demonstrated higher EBC capture capacity than commercially available solutions, which are however, standardized and easy to handle overall. On the other hand, the material of apparatus should be used with regard to minimizing the reactivity of biomarkers with condensing surface. DNA binding to the polypropylene surface of plastic labware (e.g., microcentrifuge tubes) is well documented, while the amount of absorbed DNA may range from 0.25 to 5 ng/mm^2^, depending on the ionic strength of the buffer (38). Since EBC is a fluid with low ionic strength (34), DNA-to-surface binding should not be very effective. For example, polypropylene is a hydrophobic polymer that inhibits the adsorption of the hydrophilic DNA molecule, so it is often used to make labware for DNA manipulation and storage or even for disposable EBC collection tubes. However, significant differences in DNA adsorption for different polypropylene grades and/or different surface treatments have been observed (39, 40). When considering the size of the condensation surface area, even low DNA absorption could lead to significant loss of genetic material. Thus, specialized EBC collection devices and accessories should be provided for DNA applications.

The time required for sampling is directly proportional to the volume of EBC obtained (35). Approximately 1–4 mL of the sample can be obtained in 10–25 min (Supplementary Table 1), but the resulting EBC volume varies among individuals. Inter-individual variation remains due to differences in pulmonary anatomy and resting ventilatory rates, which influence the total breath expired (32). A couple of solutions proposed to dampen this anatomical and physiological noise includes performing the collection for a time over which a pre-defined volume of breath is exhaled rather than setting a fixed collection time (28) or using visual and audio clues to control breathing patterns, such as inhalation/exhalation volumes and frequency (41). These optimizations of collection methods may improve the effectiveness of EBC biomarkers analysis.

We also hypothesize that breathing dynamics, particularly the depth of inhalation and the velocity of exhaled breath, may influence the concentration of biomarkers in EBC. Deeper inhalations are likely to draw air from more distal regions of the respiratory tract (42), increasing contact with the epithelial lining and areas where cell turnover, inflammation, or tumor activity may occur. In contrast, shallow breathing may limit sampling to the upper airways, potentially reducing the representation of lung-specific genomic material. Moreover, previous methodological studies have shown the effect of hyperventilation vs. tidal breathing on both the condensate yield and the concentration of non-volatile biomarkers such as proteins (43). Taken together, these findings suggest that breathing patterns could be a relevant pre-analytical factor in EBC collection, but further research is needed to fully elucidate their impact on the release of cfNAs into the exhaled breath.

## 3 Nucleic acid extraction

Water constitutes more than 99% of EBC, making it a liquid-based matrix in which standard body-fluid extraction kits can be applied. However, only a minor fraction of condensate derives from respiratory droplets carrying non-volatile molecules (30), so DNA yields are typically low and may not meet input requirements for downstream assays (44). A series of studies by Carpagnano et al. reported DNA yields of up to 2 μg from at least 1 mL of EBC collected with the EcoScreen device and extracted using the QIAamp DNA Mini Kit (Qiagen) (45–47). By contrast, Youssef et al. obtained an average of 75.5 ng DNA from 1.5–4.0 mL of EBC collected with the same device but processed with the QIAamp Circulating Nucleic Acid Kit (Qiagen) (48). This striking discrepancy suggests that the choice of extraction kit may account for huge variation in total DNA yield. Nevertheless, most studies summarized in Supplementary Table 1 do not report cfNA concentrations or elution volumes, preventing reliable calculation of absolute DNA recovery.

Several approaches have been tested to enhance both quality and quantity, such as sodium acetate precipitation and the use of oligo-dT primers. Yield alone has been successfully increased by SDS treatment and incubation at 70 °C (9). These adjustments nearly doubled DNA recovery (from 15.4 ± 3.6 ng/μL up to 29.3 ± 5.7 ng/μL), emphasizing the importance of pre-analytical optimization for downstream applications.

To evaluate the suitability of common EBC collection systems for downstream RNA extraction, Mehta et al. compared RTube and the Turbo-DECCS devices (49). Under identical collection and extraction conditions, RNA yields from the RTube were significantly higher, with an average of 573 ± 48 ng per 500 μL of EBC, compared to 292 ± 42 ng from the Turbo-DECCS. Among the extraction methods tested, the column-based RNeasy Micro Kit (Qiagen) consistently outperformed both the ArrayPure RNA Purification Kit (Epicenter) and TRIzol (Life Technologies) in terms of yield and consistency. Another study using the TRIzol method with EBC collected via EcoScreen, reported total RNA yields in the range of 1.8 to 2.1 μg from 500 μL of EBC across healthy, asthma, and COPD cohorts, albeit with considerable inter-individual variability (50). However, such discrepancies in RNA yield remain difficult to interpret due to insufficient reporting of pre-analytical variables and methodological details across studies.

Even two decades after the first methodological recommendations for EBC collection (30), the field continues to face the same fundamental challenge: the lack of standardized protocols to ensure reproducibility and comparability of biomarker studies, mainly those targeting cfNAs. To address this gap, we propose a Minimum Reporting Checklist for cfNA-based EBC studies (Table 2), which outlines essential methodological parameters that should be reported consistently across future investigations.

**Table 2:** Minimum reporting checklist for cfNA-based EBC studies.

**Domain**	**Parameters to report**	**Notes/Examples**
Prior to collection	Participant preparation	Abstinence of exercise, smoking, food/drink, (how long prior to collection?)
	Mouth wash^1^	Type of agent
EBC collection	Anti-saliva measures	e.g., Saliva trap, filter, swallowing instructions
	Device (commercial/custom)	Name of device; If custom device were used, condensing surface material, condensation temperature, and cooling method should be recorded
	Breathing pattern	e.g., Tidal, Deep, Controlled, Guided (visual or audio cues)
	Collection duration	Minutes
	Collection temperature	°C
	Total EBC volume	μL
EBC storage	Storage temperature	°C
cfNAs extraction	Input EBC volume	μL
	Extraction kit	Name of kit
	Modifications^1^	e.g., sodium acetate precipitation, oligo-dT primers, SDS treatment, incubation at 70 °C
	Elution volume	μL
	Nucleic acid yield	Concentration (ng/μL) or Total Quantity (ng)
	Method of quantification	Name of platform or method
cfNAs Analysis	Method	e.g., qPCR, dPCR, microarray, NGS (with platform and chemistry specified)

^1^If applicable.

## 4 Applications of cfNAs from EBC

Analyzing nucleic acids directly from EBC has shown promising potential across a range of biomedical applications (8). Breath biopsy provides a non-invasive means of investigating genetic and epigenetic alterations associated with respiratory tract diseases, including malignancies, obstructive lung diseases, and infections. Conventional diagnostic procedures, such as bronchoscopy or needle biopsy, involve procedural risks and are often impractical for routine monitoring or population-level screening. This is particularly relevant in lung cancer, where delayed diagnosis significantly reduces survival rates (51), and in chronic conditions, where repeated invasive sampling is impractical or infeasible.

As an organ-specific biofluid, EBC captures localized molecular signals from the airways that may be missed in systemic fluids like plasma, thereby complementing blood-based assays (52). Various classes of cell-free nucleic acids, including gDNA, mtDNA, mRNA, lncRNA, miRNA, and microbial nucleic acids, have been identified in EBC, each linked to specific disease contexts and potential clinical applications (Table 3). Here, we summarize these findings, outlining the diversity of cfNAs in EBC and their prospective roles in diagnosis, monitoring, and biomarker discovery.

**Table 3:** Overview of cell-free nucleic acids detected in EBC and their potential clinical applications.

**cfNAs**	**Conditions**	**Potential applications**	**Ref**.
DNA	Lung cancer	Therapy resistance monitoring (EGFR T790M mutation detection)	(52)
		Early diagnosis and screening (3p microsatellite alterations detection)	(45)
		Epigenetic biomarkers	(53, 54)
	MONs^1^ exposure	Early detection of occupational exposure-related molecular alterations (DNA methylation changes)	(55)
	Pregnancy	Prenatal screening (presence of cfDNA beyond respiratory origin)	(56)
mtDNA	COPD, Asthma	Marker of oxidative stress in obstructive lung diseases	(57)
mRNA	Lung cancer	Early diagnosis (detection of GATA6 and NKX2-1 isoforms)	(49)
miRNA	Lung cancer	Predicting clinical outcomes	(58–60)
	Asthma	Assisting endotype establishment	(61)
	Cystic fibrosis	Therapeutics targets for severe lung disease associated with chronic Pseudomonas infection; Biomarkers of pulmonary exacerbations	(62, 63)
	COPD, Asthma	Distinguishing asthma from COPD	(64)
	Tuberculosis	Diagnosis and monitoring of pulmonary and extrapulmonary TB	(65)
	Asbestos exposure	Early detection of carcinogenic exposure; Screening of high-risk populations	(66)
lncRNA	Lung cancer	Early diagnosis, patient monitoring and metastases prediction	(21)
Viral RNA	COVID-19	Diagnostics; variant monitoring	(67, 68)
Bacterial DNA/RNA	COPD	Infection monitoring in patients with acute exacerbations	(69)
	Cystic fibrosis	Microbiome profiling	(70)
Fungal DNA^2^	Asthma	Potential role in asthma severity and fungal microbiome assessment	(71)

^1^Metal oxide nanomaterials; ^2^Not yet DNA-based (culture methods used).

### 4.1 Cancer

The utility of EBC in cancer applications has advanced significantly over the past two decades. A pivotal study in 2005 demonstrated that microsatellite alterations on chromosome 3p were more frequently detected in EBC from patients with non-small cell lung cancer (NSCLC) compared to healthy controls (45). Notably, this study also showed that EBC was more sensitive than peripheral blood in detecting lung cancer-associated microsatellite alterations, highlighting its potential as a non-invasive tool for early diagnosis and screening. Building on these findings, the same group compared EBC-DNA to tumor tissue and identified matching microsatellite profiles, further reinforcing the specificity and clinical relevance of EBC-based genomic assays (46).

By 2009, promoter methylation profiling further established the role of EBC in epigenetic biomarker discovery. An early study demonstrated the technical feasibility of detecting methylation in EBC DNA and distinguished methylation patterns between smokers and lung cancer patients in key genes *DAPK, RASSF1A*, and *PAX5*β, highlighting the lung-specific origin of the signal (53). Xiao et al. subsequently focused on the tumor suppressor gene *P16*, finding frequent promoter methylation in patients with non-small cell lung cancer (NSCLC) and suggesting its potential as a diagnostic biomarker (54). Expanding the scope beyond oncology, Liou et al. assessed EBC in workers exposed to metal oxide nanomaterials. While exposure to TiO_2_, SiO_2_, and indium tin oxide (ITO) elevated oxidative stress markers such as 8-isoprostane, only ITO was linked to altered global DNA methylation (55). Together, these studies demonstrate the evolving role of EBC as a valuable source for non-invasive detection of epigenetic lung cancer-related changes and environmental exposure assessment.

In 2013, differential miRNA signatures in EBC began to emerge. The earliest report investigates expression levels of miR-21 and miR-486 in both plasma and EBC samples from NSCLC patients. This work demonstrated that miRNAs, already established as stable biomarkers in plasma, could also be detected reliably in EBC, opening the door to non-invasive molecular diagnostics via breath biopsy (58). Thereafter, a more comprehensive genome-wide miRNA profiling and machine learning analysis revealed twelve differentially expressed miRNAs between healthy individuals and lung cancer patients. Among them, miR-6777-5p, miR-6780a, and miR-877-5p demonstrated prognostic significance in predicting clinical outcomes (59). Another profiling study identified 78 overexpressed miRNAs in EBC of treatment-naive lung cancer patients compared to healthy controls. Among them, miR-31-3p, miR-449c, and let7i provide the best discriminatory power according to the authors (60).

These findings are consistent with recent systematic evaluations. A narrative review by Ferrari et al. comprehensively summarized the available evidence and concluded that EBC-derived miRNAs represent one of the most promising classes of breath biomarkers for lung cancer, with potential applications ranging from early diagnosis to monitoring of disease progression. By integrating results across multiple studies, the authors emphasized both the reproducibility of specific miRNA signatures and the need for standardized collection and analytical protocols before clinical translation can be achieved (72).

In parallel, asbestos exposure, a well-known risk factor for mesothelioma and lung cancer, was associated with distinct EBC miRNA profiles. Sequencing-based analyses demonstrated that, although miRNA concentrations in EBC were lower than in plasma, their expression patterns were consistent across both fluids. These findings suggest that EBC-derived miRNAs may capture early molecular alterations associated with carcinogenic exposures and hold promise for non-invasive screening of high-risk populations (66).

The 2016 study confirms that RNA-containing exosomes are enriched in EBC and demonstrates that, despite the relatively high fragmentation, mRNA isoforms of lung-specific transcription factors are quantifiable in EBC. Specifically, the embryonic vs. adult expression ratios of *GATA6* and *NKX2-1* were able to discriminate lung cancer patients from healthy individuals (49).

The transition from biomarker discovery toward direct genomic characterization of actionable mutations was exemplified by the first investigation of somatic mutation detection in EBC, where the EGFR T790M resistance mutation was successfully identified in patients with stage IV EGFR-positive lung adenocarcinoma. The study demonstrated that EBC may offer greater sensitivity than plasma in detecting such mutations, likely due to the acellular nature of EBC, which may contain lower levels of wild-type DNA that could otherwise mask low-frequency mutations. Moreover, the lung environment exhibits lower nuclease activity than blood, potentially enhancing the recovery of mutant cfDNA (52). Another prospective, proof-of-concept study using the UltraSEEK oncogene panel found EBC effective at identifying clinically relevant lung cancer mutations (in genes *EGFR, KRAS, PIK3CA, ERBB2, BRAF*) and suggests its complementary role with plasma-based testing in liquid biopsy lung cancer diagnostics (72). Although there was a high degree of overlap between the plasma and EBC, significant numbers of mutations occurred in one modality but not the other. This hints at the specific challenges that still remain for liquid biopsy analysis, particularly relating to consistency and validation of results. However, it also highlights the potential utility of using a combination of plasma and EBC to potentially improve diagnostic yield in liquid biopsy analysis of lung tumors.

More recently, interest has expanded toward lncRNAs as novel molecular markers in lung cancer (21). In a cohort of patients with advanced lung adenocarcinoma, the expression levels of lncRNA genes *MALAT1, HOTAIR, PVT1, NEAT1, ANRIL*, and *SPRY4-IT1* were analyzed in EBC samples. Several of these lncRNAs demonstrated potential as biomarkers for early diagnosis and patient monitoring but also for predicting the development of metastases.

Breath biopsy provides the potential to mitigate one of the most significant health concerns, the global burden of lung malignancies (73). Since the disease manifests with minimal or no obvious phenotypic changes in the early stages, the current lack of screening approaches often leads to late-stage diagnosis, allowing cancer cells to develop therapy resistance (48). So far, much research interest has been paid to blood-based liquid biopsy approaches (74), but for lung malignancies, exhaled breath represents a novel, promising source of tumor-derived DNA (75).

### 4.2 Obstructive lung diseases

Obstructive lung diseases, including chronic obstructive pulmonary disease (COPD), asthma, and cystic fibrosis, are characterized by airflow limitation, persistent inflammation, and a high susceptibility to infections. These pathological features make them ideal candidates for non-invasive monitoring by EBC, which directly captures molecular content from the lower respiratory tract.

Among the most studied nucleic acids in this context are miRNAs, which exhibit remarkable stability in various biofluids, including EBC, likely due to their encapsulation within exosomes or other extracellular vesicles, which protects them from enzymatic degradation. This intrinsic stability has enabled their use as non-invasive biomarkers in respiratory diseases. One of the earliest studies in this area identified altered expression of seven miRNAs (hsa-miR-649, hsa-miR-1264, hsa-miR-2861, hsa-miR-574-5p, hsa-miR-421, hsa-miR-624, and hsa-miR-595) in patients with asthma and tuberculosis compared to healthy individuals (76). In pediatric asthma, EBC-based profiling revealed that miR-126-3p, miR-133a-3p, and miR-145-5p were positively associated with the disease, while miR-21-5p showed a negative association with symptomatic asthma (61).

Further research focused on utilizing EBC to ascertain the differential expression of miRNAs between asthma and COPD. Sampling was conducted across three cohorts: patients with asthma, patients with COPD and a healthy control group. The comparison demonstrated exceptional discriminatory capability, with 100% differential expression observed for miR-512-3p and miR-517c, which are secreted by respiratory cells. These miRNAs can be quantified in EBC samples and employed for distinguishing between asthma and COPD (64).

In cystic fibrosis, EBC-derived miRNA signatures also proved informative. Individuals infected with *Pseudomonas aeruginosa* showed overexpression of six miRNAs (miR-1247, miR-1276, miR-449c, miR-3170, miR-432-5p, and miR-548), linked to inflammation and cell proliferation (62). Additionally, during pulmonary exacerbations in pediatric cystic fibrosis patients, four miRNAs (miR-223-3p, miR-451a, miR-27b-3p, and miR-486-5p) showed altered expression in airway samples (sputum and EBC), while no such changes were observed in blood. These miRNA changes strongly correlated with clinical outcomes reflecting exacerbation status, but seem to be restricted to the airways, highlighting the local nature of EBC-derived signals (63).

Beyond miRNAs, mitochondrial DNA (mtDNA) has also emerged as a marker of oxidative stress in obstructive lung conditions. The possibility to study mitochondrial genetic content in the EBC was investigated on patients with asthma, COPD, and asthma–COPD overlap syndrome (ACOS). In order to analyze what happens to mitochondria, both locally and systemically, mtDNA-to-nuclear DNA (nDNA) ratio was quantified in paired blood and EBC samples. Elevated mtDNA/nDNA ratios were observed in COPD and ACOS, with a rising trend in asthma, compared to healthy controls. Moreover, a positive correlation between EBC and blood ratios suggests that EBC could reflect both local and systemic mitochondrial dysfunction. These findings support the feasibility of using mtDNA in EBC as a non-invasive marker of oxidative stress, although validation in larger cohorts is still needed.

### 4.3 Infections

Several respiratory diseases are associated with dysbiosis in the pulmonary microbiome, which can influence disease onset, progression, or exacerbation. By analyzing microbial nucleic acids in EBC, breath biopsy may enable early detection of such imbalances, offering insights into conditions like asthma, COPD, cystic fibrosis, and even lung cancer. Early efforts were focused on detecting microbial DNA and RNA in patients with acute exacerbations of COPD. Bacterial or viral nucleic acids were identified in EBC and sputa, using species-specific PCR assays. Interestingly, the results from EBC did not correlate well with those from sputum, indicating both technical feasibility and the need for methodological refinement (69).

Comparative microbiome analyses further characterized the potential and limitations of EBC as a sampling medium. In an animal model study, microbial DNA yields from EBC were significantly lower than those from protected specimen brushing, and microbial community structures differed between the two sample types, suggesting that EBC may reflect a distinct airway niche or be influenced by sampling depth (70). Although EBC is not recommended as a replacement for more invasive techniques due to low microbial biomass and susceptibility to contamination, it remains a promising option for studies where non-invasive sampling is essential. Further research into the mechanisms of EBC formation and the factors influencing its composition is warranted to better interpret its diagnostic potential.

The COVID-19 pandemic notably accelerated the clinical utility of breath biopsy for infectious disease diagnostics. Initial studies demonstrated that RT-PCR performed on EBC could reliably detect SARS-CoV-2 originating from the lower respiratory tract and correlated with disease severity (67). Viral RNA levels in EBC peaked within 2 days of symptom onset and declined among non-ventilated individuals thereafter. On the other hand, in mechanically ventilated patients, detection rate and viral load were high regardless of days since onset (77). A follow-up study further demonstrated that the Delta variant exhibited significantly higher EBC viral loads compared to the wild-type strain, supporting the feasibility of EBC-based diagnostics in highly transmissible variants and early-phase infections (68).

The potential of EBC for microbiome assessment extends beyond bacterial and viral pathogens. In asthma patients, Carpagnano et al. investigated the presence of fungi in both EBC and sputum samples using a culture-based detection method. Fungal colonization patterns were consistent across the two sample types and were associated with more severe asthma, persistent symptoms, and reduced disease control. Although DNA-based methods were not used, these findings suggest that fungal DNA may indeed be present in EBC, warranting further molecular studies to explore the potential of EBC for fungal microbiome analysis (71).

Beyond microbial DNA and RNA, EBC miRNAs have also been investigated in the context of infectious diseases. In tuberculosis, qRT-PCR-based profiling revealed significant upregulation of miR-454, miR-139, and miR-143 in EBC, suggesting their potential as non-invasive biomarkers for both pulmonary and extrapulmonary tuberculosis. These findings provide early evidence that miRNA signatures in EBC could complement conventional microbiological assays in the diagnosis and monitoring of respiratory infections (65).

Together, these studies establish EBC as a valuable and non-invasive fluid for investigating respiratory infections of bacterial, viral, and fungal origin. While sensitivity and concordance with conventional sampling methods may vary, the organ-specific nature of EBC and its suitability for repeat sampling highlight its promise for future use in clinical microbiology, outbreak monitoring, and respiratory pathogen surveillance.

### 4.4 Non-organ-specific EBC applications

Much has been written about the presence of cfDNA in EBC in relation to respiratory tract diseases, but DNA originating from tissues not in direct contact with the airways remains poorly investigated. Recently, the presence of cell-free fetal DNA in maternal EBC was demonstrated, suggesting that EBC may not only sample the respiratory system but also reflect genetic material from the entire body. Despite its low detection rate, the mechanism by which cfDNA from distant tissues enters respiratory droplets warrants further investigation to fully uncover its potential in biomedical applications (56).

## 5 Methodological considerations for cfNA analysis in EBC

The detection of cfNAs in EBC has been approached using a variety of analytical platforms, each with advantages and limitations. For DNA, conventional PCR and qPCR assays have been applied to detect specific mutations, microsatellite alterations, or methylation changes (44, 45, 54). Digital PCR (dPCR) has further improved sensitivity and quantitative accuracy, enabling detection of rare alleles at low frequencies, although it remains limited to targeted loci (52). More recently, targeted next-generation sequencing (NGS) panels have been explored in proof-of-concept studies to identify clinically relevant mutations in EBC DNA, with results suggesting a potential complementary role alongside plasma-based liquid biopsy (72).

For RNA species, including mRNA, lncRNA, and particularly miRNAs, qRT-PCR remains the most widely used technique because of its sensitivity and specificity for predefined targets (61, 63). While qRT-PCR is considered a reliable standard, it is limited in its ability to detect novel or unknown miRNAs (58, 65, 78). Microarrays have also been used as cost-effective tools for high-throughput exploratory profiling, enabling simultaneous analysis of numerous RNA targets (59). However, its lower sensitivity and specificity compared to qRT-PCR or NGS limit the utility for low-abundance transcripts. Increasingly, NGS is regarded as the preferred platform, as it allows both quantification of known transcripts and discovery of novel molecules, a critical advantage in light of the typically low RNA yields associated with EBC (58, 66, 79). NGS provides the most comprehensive resolution and is likely to dominate future cfNA studies in breath biopsy. However, the higher cost of this technique remains a factor in method selection (66, 80).

## 6 Conclusions

Although it is traditionally underutilized compared to plasma, EBC is increasingly recognized as a promising, organ-specific fluid for non-invasive molecular diagnostics. Our review demonstrates that diverse classes of cfNAs, including genomic DNA, mitochondrial DNA, RNA species, and microbial genetic material, can be recovered from EBC and linked to clinically relevant applications in lung cancer, obstructive lung diseases, and infections. However, standardization remains one of the major barriers to clinical translation, thus we propose a Minimum Reporting Checklist for cfNA-based EBC studies, emphasizing consistent reporting of collection, extraction, and analytical variables. Despite other technical challenges such as low nucleic acid yield and contamination risk, EBC offers distinct advantages, including repeatable, entirely non-invasive sampling, localized airway specificity, and compatibility with portable devices that enable home collection. This user-friendly approach may encourage more frequent preventive testing and help reduce medical care avoidance, thereby improving early disease detection and patient outcomes.

Looking forward, several trends are likely to shape the field, including miniaturized and wearable condensers for real-world sampling or harmonized breathing protocols to reduce variability. As one of the current trends in liquid biopsies, fragmentomic and epigenomic profiling may also extend the informative value of breath biopsy beyond mutation detection. Hand in hand, instead of focusing on individual variants, genome-wide strategies are emerging that can capture broader genomic patterns from minimal amounts of cfDNA. Multimodal approaches may provide more robust signals in low-yield samples and generate multidimensional datasets, where artificial intelligence can uncover diagnostic patterns. Finally, large-scale prospective studies, ideally including head-to-head comparisons with plasma, are needed to validate the clinical value of EBC-based assays.

Ongoing research is expected to improve the sensitivity and reproducibility of EBC-based assays, expand their application beyond respiratory diseases, and integrate them into multimodal diagnostic strategies. Although that may seem like a distant perspective in general healthcare, with continued optimization, breath biopsy may soon transform from an experimental technique into a clinically viable tool for early disease detection, longitudinal monitoring, and population-level screening.
